# Microgrooves with Small Taper Angle Processed by Nanosecond Laser in Closed Flowing Water

**DOI:** 10.3390/mi15040448

**Published:** 2024-03-27

**Authors:** Guoyan Chen, Junfei Zhang, Jian Yuan, Bin He, Jinjin Han, Suorong Zhang

**Affiliations:** 1 Engineering Training Center, Jiangsu University of Technology, Changzhou 213001, China; jxcgy@jsut.edu.cn; 2 School of Mechanical Engineering, Jiangsu University of Technology, Changzhou 213001, China; zjf18226596755@163.com (J.Z.); yj250894104@163.com (J.Y.); hjj052@jsut.edu.cn (J.H.); zhangsr@jsut.edu.cn (S.Z.)

**Keywords:** underwater laser processing, bubble, flowing water, taper angle

## Abstract

To improve the capability of nanosecond lasers to process structures with a high aspect ratio, a new method of nanosecond laser processing in closed flowing water was proposed in this paper. The microgrooves on a stainless steel 304 surface were processed by the new method, and the influence of processing parameters on the microgrooves was studied. The comparative experiments of laser processing in still water and overflowing water were also carried out, and the unusual phenomenon of laser processing in different flowing water was discovered by a high-speed camera. The results showed that the flowing velocity played a crucial role in underwater laser processing, and that high flowing velocity could timely remove bubbles in closed flowing water, thus obtaining higher processing efficiency. As the depth of the groove increased, the bubbles firstly affected the processing of the sidewall, causing a circular transition between the sidewall and bottom surface. The reflection of the laser beam by the bubble could cause secondary processing on the sidewall, resulting in a decrease in the taper angle. Based on the above research, the microgroove with a width of 0.5 mm, aspect ratio of 3, and taper angle of 87.57° was successfully processed by a nanosecond laser in closed flowing water. Compared to conventional nanosecond laser processing, laser processing in closed flowing water was more advantageous in processing microgrooves with a small taper angle and high aspect ratio.

## 1. Introduction

Laser processing, as a non-traditional machining method that relies on laser energy to remove materials [1], has advantages of high machining efficiency and high flexibility, regardless of material strength and hardness [2], and is widely used in the processing of difficult materials such as stainless steel, titanium alloy, ceramics, and various composite materials [3,4,5]. A short pulse laser (nanosecond pulse laser) and ultra-short pulse laser (picosecond and femtosecond pulse laser) can realize the processing of microstructures due to their narrow pulse width, high energy density, and short action time with materials [6,7,8,9,10].

In laser processing, the existence of a heat-affected zone and the accumulation of products are the main factors affecting the surface quality and machining accuracy, which lead to the formation of a recast layer and the taper angle [11,12,13]. To reduce the heat-affected zone and recast layer, laser ablation is carried out in liquids to utilize the cooling effect of the liquid. Owing to their high thermal conductivity, non-toxicity, and easy availability of water, underwater laser processing methods, mainly including those in still water and flowing water, are the most commonly used methods in the laser ablation of several materials [14,15,16].

Bubble formation is an inevitable phenomenon that is observed in underwater laser processing, which is related to the vaporization of a portion of water caused by the high laser-induced pressure and temperature of plasma [17,18,19]. The stability of underwater laser processing was relatively poor due to the complex interaction between the laser beam, bubbles, and ablated particles, especially in still water. In flowing water laser processing, the flowing water can promote a flushing action for the removal of bubbles and ablated products, providing a more relatively steady hydrodynamic than that in the still water [20,21]. Therefore, the laser processing in flowing water is more preferred for precision microstructures than that in still water. Tangwarodomnukun et al. [22] used a low-pressure waterjet to create a thin and flowing water layer on the workpiece surface, and the laser beam is applied for ablating the material within the boundary of the flowing layer. The results showed that flowing water could significantly diminish the optical disturbance usually found in the still water method. Zhou et al. [23] studied the laser ablation of silicon under the thin flow water layer. The results showed that the velocity of water flow and pressure had important effects on the processed depth and surface morphology, and that the larger velocity and pressure is conductive to process smooth grooves.

Although laser processing in flowing water has been extensively studied, shallow structures of only tens of microns were usually processed in previous research. The process of material removal and shape contour change was not clearly revealed. To realize the processing of deep and straight structures, a device with a closed flow path was designed for laser processing in closed flowing water in this paper, and a cost-effective nanosecond laser was used to process the microgroove structures on a stainless steel surface. Different from other studies, this paper focused on the change in the contour and sidewall taper angle of the grooves processed in flowing water. The optimum parameters of laser processing in closed flowing water were first obtained by analyzing the cross-section contour of the grooves, and the experiments of different processing times were conducted with the optimal parameters to reveal the evolution process of the contour of the groove. As a contrast, the grooves were also processed in still water and overflowing water, and the experimental phenomena in three different water conditions were observed by high-speed cameras. Combined with the observed bubble distribution in experiments and the processed results of the grooves, the laser processing under different conditions was discussed. Finally, the new method of this study was applied to the processing of microgrooves with a large depth and small sidewall taper angle.

## 2. Experiments’ Design

### 2.1. Method of Laser Processing under Different Water Conditions

**Figure 1:**
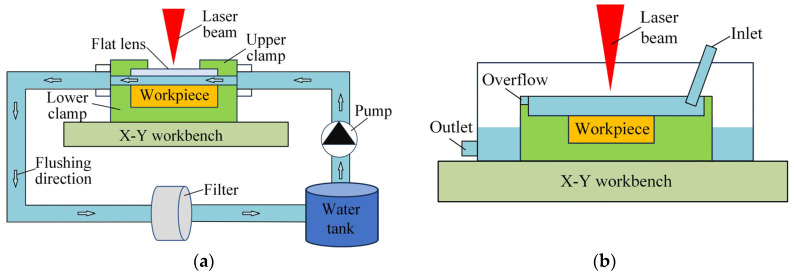
Experimental equipment of laser processing under different conditions. (**a**) In closed flowing water. (**b**) In open overflowing water.

As shown in Figure 1a, a new fixture was designed to realize laser processing in closed flowing water, which was composed of the upper clamp and the lower clamp. The upper clamp was provided with a window for laser processing, and a glass sheet with a laser transmittance of over 99% was installed at the window. The lower clamp was designed with a flow channel, in which the workpiece was installed. The length of the flow channel was 100 mm, the width was 30 mm, and the depth was 2 mm. The upper surface of the workpiece was leveled with the bottom surface of the flow channel, and the upper surface of the flow channel was leveled with the bottom surface of the glass. In laser processing, the clean water was pumped from the storage tank into the fixture, the laser beam through the glass, and the flowing water to the workpiece surface, achieving the processing of the workpiece material. The flowing water carried the products and bubbles out of the processing area, and then passed through the filter to remove the products. Finally, the filtered water returned to the storage tank for recycling. In experiments, the output flow of the pump could be adjusted freely through its own valve, and the maximum output flow rate was 14.5 L/min, corresponding to a flow velocity of about 4 m/s in the processing zone. The polypropylene (PP) filter with a resolution of 5 μm was used, and the water was replaced after processing for about 2 h or after completing a long processing experiment.

As shown in Figure 1b, laser processing in overflowing water was carried out in an open environment. The water flowed from one end of the fixture into the processing area, and flowed out from the other end through the overflowing port. The thickness of the water layer above the workpiece surface was controlled to be 2 mm by changing the size of the overflowing port. The device could also be used for laser processing in still water when the fixture was filled with water and the inlet was closed.

The laser processing experiments were conducted on the laser marking machine equipped with a nanosecond fiber laser (MDK-GX-20Z, Suzhou, China); the wavelength of the laser was 1064 nm, the pulse width was 100 ns, and the beam spot size was 0.05 mm. Due to the influence of the water layer, the laser spot was focused below the initial surface of the workpiece, which was the negative off-focus. In experiments, the negative off-focus of 1 mm was used. The stainless steel 304 workpieces were used in experiments, its density was 7.93 g/cm^3^, the melting point was 1398–1452 °C, and the thermal conductivity was 16.3 W/m·K. The workpiece size was 20 mm (width) × 30 mm (length) × 2 mm (thickness), and multiple experiments could be conducted on each workpiece. The precision X-Y platform was used to ensure that the processing area was in the center of the galvanometer in each experiment.

### 2.2. Experimental Design

To clarify the influence of the processing parameters on the laser processing in closed flowing water, orthogonal experiments were carried out. The three parameters of laser power, path interval, and scanning speed were considered, with four different levels of values selected for each parameter. Therefore, a total of sixteen experiments were conducted, corresponding to the level of L1–L16 in Table 1. In each experiment, the flow velocity of water was 4 m/s, and the groove of 1 mm × 3 mm was processed for 1000 times. The processed grooves were ground from the side to observe the cross-section by scanning electron microscopy (SEM, sigma 500, Zeiss, Germany), and the taper angle of the sidewall was measured by laser scanning confocal microscopy (LSCM, VT6000, Chotest, Shenzhen China) to optimize the processing parameters.

Using the optimum parameters obtained from orthogonal experiments, the grooves were processed for different times. The sidewall contour of the processed grooves was extracted to analyze the evolution during the laser processing in closed flowing water. In addition, the grooves with different depths were processed in still water and overflowing water, and the cross-section of the processed grooves was also detected. The experiment phenomenon of laser processing in still water, overflowing water, and closed flowing water was observed by high-speed cameras (M220, Revealer, HeFei, China). Finally, the microgrooves with a high aspect ratio were processed to verify the feasibility of nanosecond laser processing in closed flowing water.

## 3. Results

### 3.1. Orthogonal Experiment Results of the Laser Processing in Closed Flowing Water

**Figure 2:**
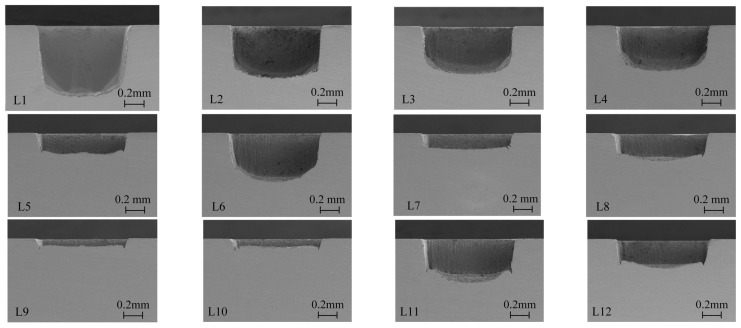

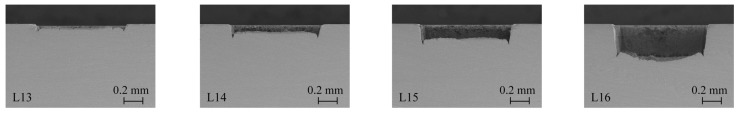
The cross-section morphology of grooves obtained by orthogonal experiments.

The orthogonal experimental results of different processing parameters are shown in Figure 2, and it can be found that the sidewall contour and the groove depth were different with different processing parameters. Under the same processing times, the groove depth of L13 was less than 0.1 mm, while the groove depth of L1 was more than 1 mm. For the shallower grooves (L7, L9, L10), the bottom surface was relatively flat. However, the bottom surface of the deeper grooves (L1, L2) appeared to be of a circular shape. Moreover, it can be observed that the sidewalls of the deep grooves (L2, L3) are more vertical than those of shallow grooves (L7, L9, L10).

**Figure 3:**
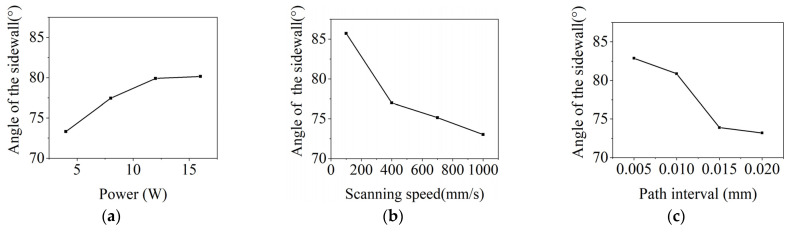
Taper distribution of groove sidewall processed with different parameters. (**a**) Laser power. (**b**) Scanning speed. (**c**) Path interval.

The taper angle of the groove sidewall is an important indicator for evaluating processing quality. To reveal the influence of the laser power, scanning speed, and path interval on the groove structure, the taper angle of the groove sidewall processed with orthogonal experiments was analyzed. The larger taper angle corresponds to the steeper sidewall, which means a higher processing quality. Therefore, the values corresponding to the maximum taper angle of the sidewall for each parameter was the optimal. As shown in Figure 3, with the increase in laser power and the decrease in the path interval and scanning speed, the taper angle gradually increased. Therefore, a high laser power, small scanning speed, and path interval were helpful in achieving the high processing quality of the groove. As a result, a laser power of 16 W, scanning speed of 100 mm/s, and path interval of 0.005 mm were selected as the optimal parameters for the following lasing processing experiments.

### 3.2. The Evolution of Grooves Processed by Nanosecond Laser under Different Conditions

**Figure 4:**
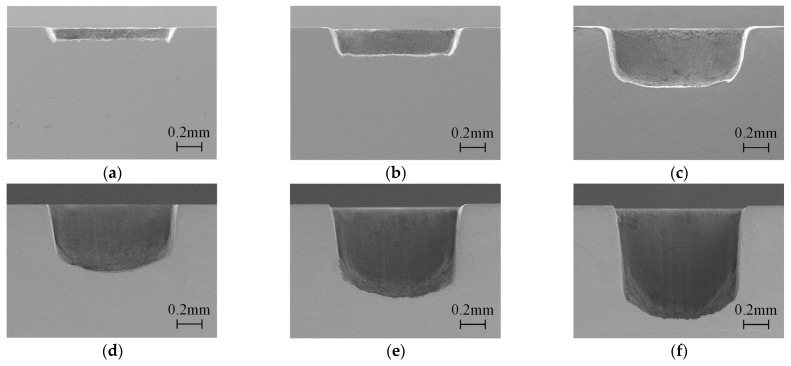
The cross-section morphology of the grooves with different processing times in closed flowing water: (**a**) 50 times, (**b**) 100 times, (**c**) 200 times, (**d**) 400 times, (**e**) 600 times, and (**f**) 800 times.

**Figure 5:**
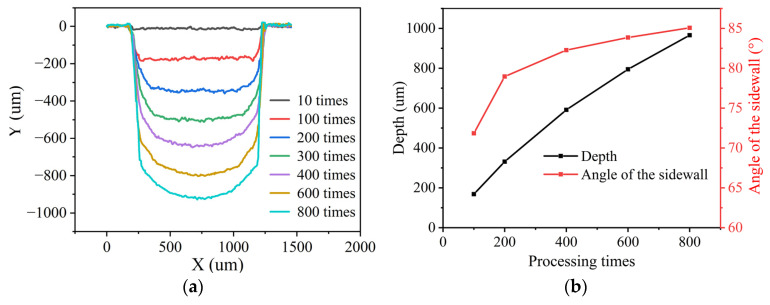
Results of grooves processed with different times in closed flowing water. (**a**) Contour of cross-section. (**b**) Depth and sidewall taper angle.

To study the forming process of the groove processed in closed flowing water, experiments with different processing times were carried out by using the optimal parameters obtained above, and a flow velocity of 4 m/s was used in each experiment. The results are shown in Figure 4 and Figure 5. It can be observed that the depth of the grooves increased significantly with the processing times. As shown in Figure 4a,b, when the depth of the groove was shallow, the bottom surface of the groove was flat, and there was no transition between the sidewall and bottom surface. As the depth of the groove increased, the arc transition occurred between the sidewall and the bottom surface of the grooves, as shown in Figure 4c. When the number of processing times was greater than 400 times, the bottom surface of the groove developed into an arc, as shown in Figure 4d–f. It can be seen from Figure 5 that the angle of the sidewall increased along with the depth of the grooves, indicating that the sidewall became increasingly vertical. For the groove processed 800 times, its maximum depth was 0.97 mm, and the taper angle of the sidewall was 85.07°.

**Figure 6:**
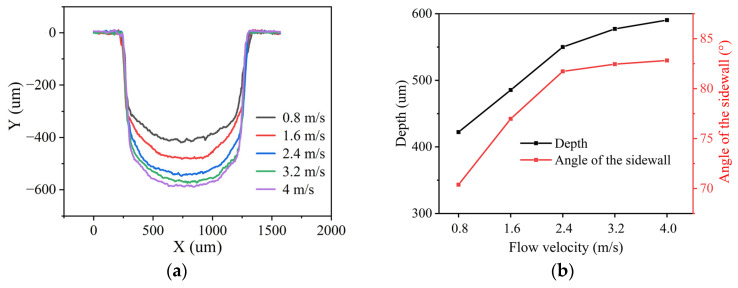
Grooves processed at different flow velocities in closed flowing water. (**a**) Contour of cross-section. (**b**) Depth and sidewall taper angle.

To study the effect of the flow velocity of water on laser processing in closed flowing water, experiments in closed flowing water were conducted under different flow velocities. As a low flow pump was used in experiments, the flow velocity of 0.8 m/s, 1.6 m/s, 2.4 m/s, 3.2 m/s, and 4 m/s was selected. The number of processing times was uniformly 400 times. The cross-section contour, depth, and sidewall taper angle of the grooves processed at different flow velocities were measured, and the results are shown in Figure 6. It can be found that the depth and sidewall taper angle were both increased with the increase in the flow velocity of water. When the flow velocity was less than 2.4 m/s, the depth and taper angle increased significantly with the flow velocity, indicating that the change in the flow velocity of water had a great impact on the laser processing. When the flow velocity was greater than 2.4 m/s, the depth and taper angle increased less with the flow velocity, meaning that the change in flow velocity had less of an influence on the laser processing.

**Figure 7:**
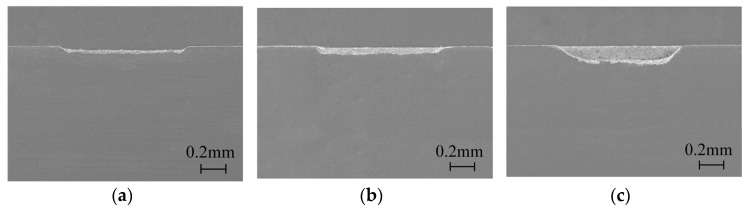
The cross-section morphology of the grooves processed with different times in still water: (**a**) 100 times, (**b**) 200 times, (**c**) 400 times.

As a comparison, the forming processes of the grooves processed by a nanosecond laser in still water and overflowing water were also studied. The grooves of different depths were processed with the same parameters of 16 W, 100 mm/s, and 0.005 mm. In still water laser processing, the initial depth of water was 2 mm, which was the same as the depth of a channel in closed flowing water. Due to a lack of supplementation and evaporation under laser action, the water was continuously reduced in still water laser processing. As a result, the processing times in still water were far less than those in closed flowing water, and the processing times of 100 times, 200 times, and 400 times were selected in still water laser processing. As shown in Figure 7, it can be easily observed that the depth and contour of the grooves processed in still water were much worse than those in closed flowing water. The depth was 0.05 mm, 0.08 mm, and 0.13 mm, corresponding to 100 times, 200 times, and 400 times. It can be assumed that the laser was difficult to remove material in still water.

**Figure 8:**
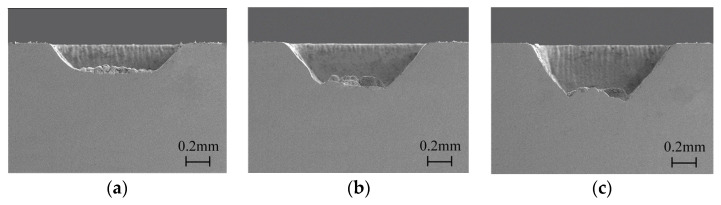
The cross-section morphology of the grooves processed with different times in overflowing water: (**a**) 500 times, (**b**) 800 times, (**c**) 1000 times.

As shown in Figure 8, the depth of the grooves processed in overflowing water increased with processing times, which was 0.21 mm, 0.36 mm, and 0.42 mm, corresponding to 500 times, 800 times, and 1000 times, respectively. Obvious recast products could be observed at the bottom surface of the grooves, and the sidewall of the grooves was more inclined than that processed in closed flowing water. It can be concluded that the quality of the grooves processed in overflowing water was worse than that processed in closed flowing water.

### 3.3. Observation of Laser Processing under Different Conditions

**Figure 9:**
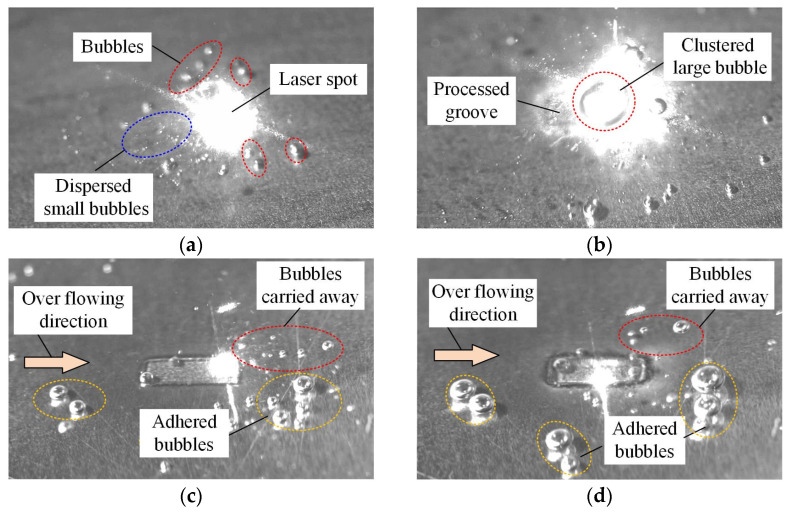

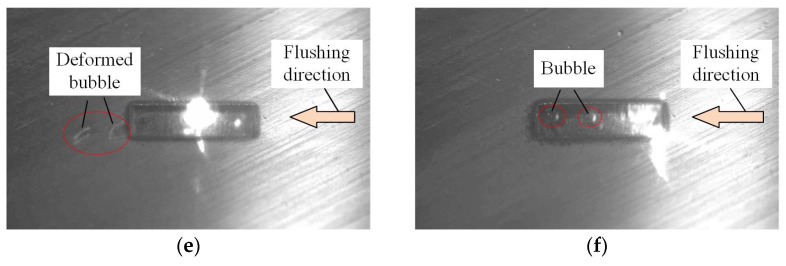
Experimental phenomena observed by high-speed cameras in different laser processing: (**a**) 1st processing time in still water, (**b**) 50th processing time in still water. (**c**) 100th processing time in overflowing water, (**d**) 400th processing time in overflowing water, (**e**) 100th processing time in closed flowing water with high velocity of 4 m/s, (**f**) 400th processing time in closed flowing water with high velocity of 4 m/s.

In this section, the three laser processing processes in still water, overflowing water, and closed flowing water were observed by a high-speed camera, in which grooves of 1 mm × 3 mm were processed. As shown in Figure 9a, even at the initial stage of laser processing in still water, the bright area generated by the laser beam was larger than the groove, numerous dispersed small bubbles were thrown out from the bright area, and larger bubbles existed further away from the bright area. When the processing times reached 50 times, the bright area shown in Figure 9b was much larger than the first time, and clustered large bubbles appeared in the center of the bright area. This is because that although some bubbles were thrown out of the processing area by the plasma explosion, some of the bubbles generated around the processing area moved towards the water surface under the function of buoyancy. These bubbles could not be taken away in still water, and a large number of bubbles were in the water, which reflected the laser beam, making the observed bright area significantly larger than the processing area. With the increase in processing times, the bubbles in the water increased and clustered into large bubbles, the reflection effect of the laser beam was enhanced, and the bright area became larger than the initial stage.

For the laser processing in overflowing water shown in Figure 9c, the bright area was about the same as the width of the groove, which was smaller than that in still water. The dispersed small bubbles and relatively large bubbles produced by laser processing were carried away along the direction of overflowing, but some relatively large bubbles accrued in the groove and moved to the opposite direction of the overflowing. In addition, some bubbles adhered to the workpiece surface far from the processing area and were not carried away by the overflowing water, which were marked as the adhered bubbles in Figure 9c,d. With the increase in processing times, as shown in Figure 9d, larger bubbles appeared in the groove and moved to the opposite direction of the overflowing, and the adhered bubbles became larger. Compared with the processing in still water, the overflowing of water could carry most bubbles away from the processing area, reducing the reflection of the laser beam. However, due to the low overflow velocity of the water, there were still a small number of bubbles in the processing area, which had an impact on the laser processing.

The experimental phenomenon of the laser processing in closed flowing water with a high velocity is shown in Figure 9e,f. When the processing time was 100 times, the bubbles produced by the laser beam were quickly carried away and deformed under the impact of flowing water, and bubbles were invisible in the groove. As the processing time increased to 400 times, bubbles were observed in the groove, which were fewer than those in the overflowing water. Therefore, it can be inferred that laser processing in closed flowing water was superior to the laser processing in overflowing water.

## 4. Discussion

From the above experimental results, it can be found that the processing depth and sidewall taper angle was different under different conditions of water, corresponding to that a different processing efficiency and quality was obtained with different underwater laser processing. Laser processing in still water had the worst efficiency and quality. For laser processing in overflowing water, the depth increased, but the quality was still poor. When processed in closed flowing water of a high flow velocity, the processing efficiency and quality had been significantly improved, and grooves that were deep and straight could be processed. The above conclusions were consistent with the existing studies, which can be explained as high-speed flowing water could reduce the impact of bubbles on laser processing to obtain better surface roughness and smaller heat-affected zones [12,21,23]. However, the existing studies have not clearly presented the effect of bubbles on the processing contour and sidewall taper angle.

In this study, the distribution of bubbles was directly observed by a high-speed camera and a detailed explanation of the experimental results was given combined with the distribution of bubbles. In still water, the bubbles generated during processing were all in the water, and increased with the processing time. The laser beam was seriously shielded by a large number of bubbles in the water, leading to poor localization and low material removal. As a result, shallow and low-quality grooves in Figure 7 were processed in still water. In overflowing water, some of the bubbles generated by a laser beam were carried away. The shielding effect on the laser beam was weaker than that in still water, so that the depth of grooves increased. However, due to the water flow velocity being relatively low, the processing was still affected by bubbles. Consequently, the poor grooves in Figure 8 were processed in overflowing water.

**Figure 10:**
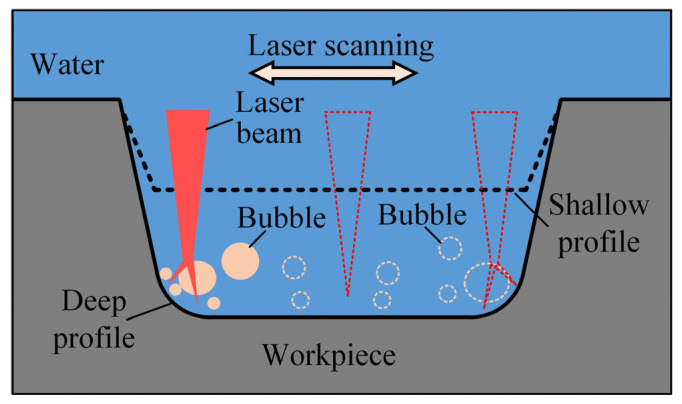
Schematic diagram of microgroove processed by nanosecond laser in closed flowing water.

In closed flowing water, when the processing times were less, the groove was shallow and the flowing rate of water inside the groove was high. Therefore, bubbles could be quickly removed from the processing area, the processing was almost unaffected by bubbles, and a deep groove with a flat bottom was obtained. As the processing depth of the grooves increased, the flowing velocity of the water inside the groove decreased, especially near the sidewall, and the bubble firstly affected the processing of the sidewall. As shown in Figure 10, the bubble near the sidewall could shield and reflect the laser beam, which weakened the processing of the bottom and produced secondary processing on the sidewall, causing the two ends of the groove bottom to become arcs. With the processing times increased, the entire bottom of the groove developed into arcs.

**Figure 11:**
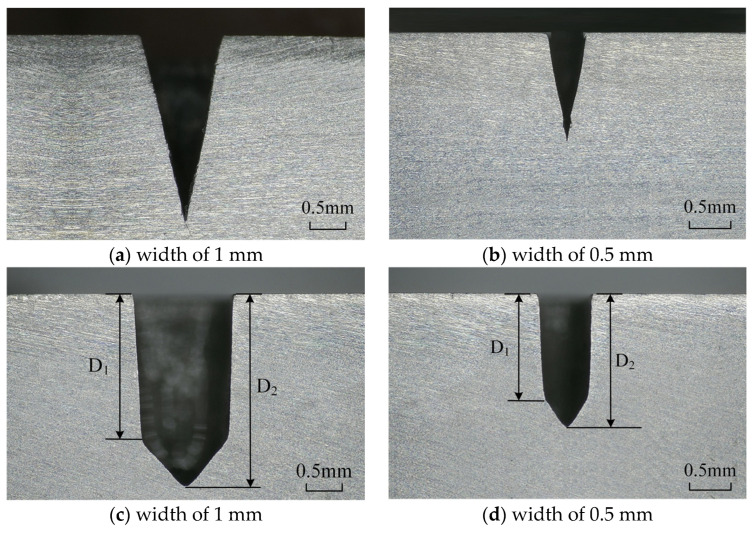
Deep microgroove structures processed by nanosecond laser, (**a**,**b**) in air, (**c**,**d**) in closed flowing water.

To further demonstrate the machining capability of laser processing in closed flowing water, deep microgroove structures with different widths were processed by a nanosecond laser in air and closed flowing water. The experiments of laser processing in air were conducted with the optimal parameter, laser power of 16 W, scanning speed of 500 mm/s, and path interval of 0.01 mm. As shown in Figure 11a,b, the bottom of the microgrooves processed in air was sharp, and the sidewall taper angle was large. The depth and taper angle of the microgrooves with a width of 1 mm and 0.5 mm were 2.45 mm, 75.38°, and 1.49 mm, 79.41°, respectively. The deep microgrooves processed in closed flowing water were better than that processed in air. For the microgroove with a width of 1 mm (Figure 11c), the deep D_1_ and D_2_ was 2.03 mm and 2.62 mm, and the taper angle of D_1_ was 86.87°. For the microgroove with a width of 0.5 mm (Figure 11d), the deep D_1_ and D_2_ was 1.48 mm and 1.88 mm, the corresponding aspect ratio was 2.96 and 3.76, and the sidewall taper angle of D_1_ was 87.57°. Therefore, compared to conventional laser processing, laser processing in closed flowing water was more advantageous in processing microgrooves with a small taper angle and high aspect ratio.

## 5. Conclusions

In this paper, a new method of laser processing in closed flowing water was presented and applied to the nanosecond laser processing of microgrooves with a small taper angle. A series of experiments with different parameters were carried out, and the forming process of a microgroove processed by a nanosecond laser under different water conditions was analyzed. The specific conclusions are summarized as follows:

(1) For the nanosecond laser processing of stainless steel in closed flowing water, a high laser power, low laser scanning speed, and small laser path interval were more preferred to obtain the microgroove structure with a large depth and straight sidewall.
(2) The flow state of water had an important impact on underwater laser processing. Compared with laser processing in still water and overflowing water, the laser processing in closed flowing water was less affected by bubbles and had higher processing efficiency and quality.
(3) High-quality microgrooves could be processed by a nanosecond laser in closed flowing water; for the groove with a width of 0.5 mm, the aspect ratio could reach three, and the taper angle was 87.57°.

## Figures and Tables

**Table 1:** Parameters of orthogonal experiments.

Level	Scanning Speed/mm·s^−1^	Path Interval/mm	Laser Power/W
L1	100	0.005	4
L2	100	0.010	8
L3	100	0.015	12
L4	100	0.02	16
L5	400	0.01	4
L6	400	0.005	8
L7	400	0.02	12
L8	400	0.015	16
L9	700	0.015	4
L10	700	0.02	8
L11	700	0.005	12
L12	700	0.01	16
L13	1000	0.020	4
L14	1000	0.015	8
L15	1000	0.01	12
L16	1000	0.005	16

## Data Availability

The original contributions presented in the study are included in the article, further inquiries can be directed to the corresponding author.
